# Supplementary material for: Emergence and maintenance of modularity in neural networks with Hebbian and anti-Hebbian inhibitory STDP
Source: PLoS Comput Biol. 2025 Apr 22;21(4):e1012973. doi: 10.1371/journal.pcbi.1012973 (PMC12054933; doi:10.1371/journal.pcbi.1012973)
Supplement: S1 Text — (PDF) [file pcbi.1012973.s001.pdf]

S1 Text. Untrained group of neurons.

This alternative protocol is analogous to the one of the numerical experiment reported in Fig 1D of the main text. The only difference lies in the fact that a group of excitatory neurons is never stimulated and so it is untrained. The results obtained are described in Fig A. On the one hand, we obtain analogous results with the formation of two modular structures in the weighted connectivity associated to spontaneous recalls of the two different memories during the dynamical evolution as shown in raster plot. On the other hand, neurons of the untrained group are weakly connected among them in accordance with the absence of stimulation and are decoupled from the other clusters while receiving anti-Hebbian inhibition from them. As a result, these neurons spike in a totally asynchronous and irregular way, without impacting the dynamics of the rest of the network.

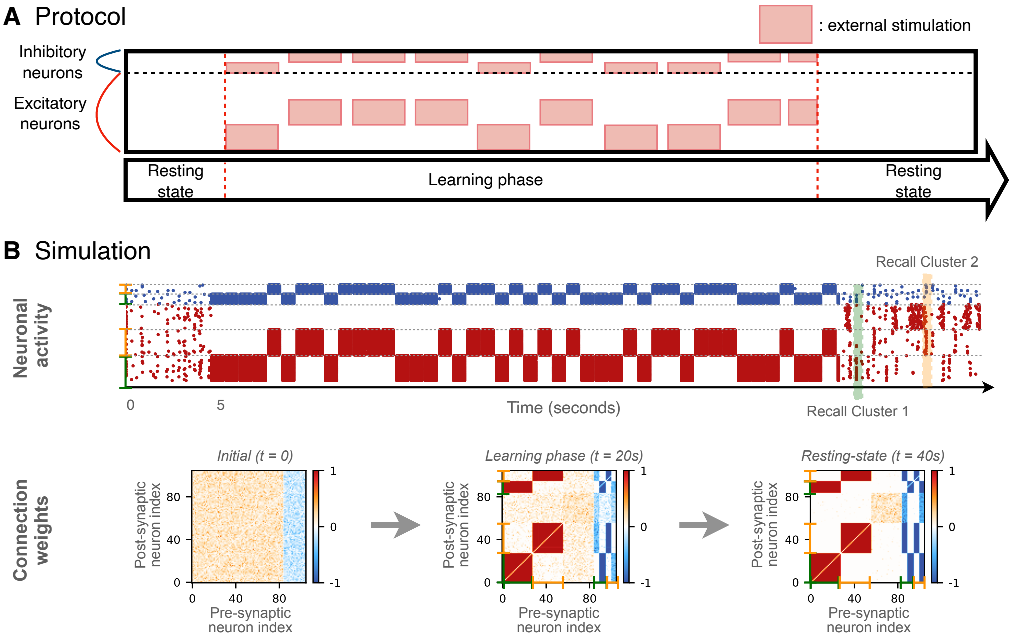

Fig A. Learning of 2 stimuli with an untrained sub-population. (A) Stimulation protocol for a network of $N = 105$ neurons entrained with $M = 2$ stimuli with an untrained group of excitatory neurons. **(B)** Simulation and learning results. Connectivity matrices show the evolution of the synaptic weights leading to the emergence of two modules and the decoupling of the unstimulated sub-population. The raster plot shows the simulation for the three stages: initial resting phase, entrainment stage and the post-learning neuronal activity characterized by spontaneous recall events of P_1 neurons (green shadow) and P_2 neurons (orange shadow).
