# Supplementary material for: Emergence and maintenance of modularity in neural networks with Hebbian and anti-Hebbian inhibitory STDP
Source: PLoS Comput Biol. 2025 Apr 22;21(4):e1012973. doi: 10.1371/journal.pcbi.1012973 (PMC12054933; doi:10.1371/journal.pcbi.1012973)
Supplement: S2 Text — (PDF) [file pcbi.1012973.s002.pdf]

S2 Text. Randomly stimulated neurons within each population.

This alternative protocol is analogous to that of the numerical experiment shown in Fig 1D of the main text. The only difference lies in the fact that when a population is selected during learning, a random number of neurons in the excitatory population (with a probability of 0.5) is stimulated. The results obtained are described in Fig A. The direct consequence is that the two modular structures in the weighted connectivity are less well formed compared to the original experiment. Nevertheless, the clusters remain decoupled with the same feedforward and feedback inhibition described in the main text. Only the weights within the clusters appear have more random values. Therefore, although the spontaneous recalls of the two memory items are present in the dynamics as shown in the raster plot, they appears to be somewhat sparser and less synchronized. We assume that this is largely due to the fact that the connections to and from the inhibitory neurons are incomplete. In Fig 3A of the main text, the randomness of the E-E connections does not impact the spontaneous recalls. This highlights the need to reach a convergence of the weights linked to inhibition to correctly memorize the items. Nevertheless, this experiment also shows that even if memory items are partially learned during each period of stimulation, the entire original memory item is somehow retrieved and learned.

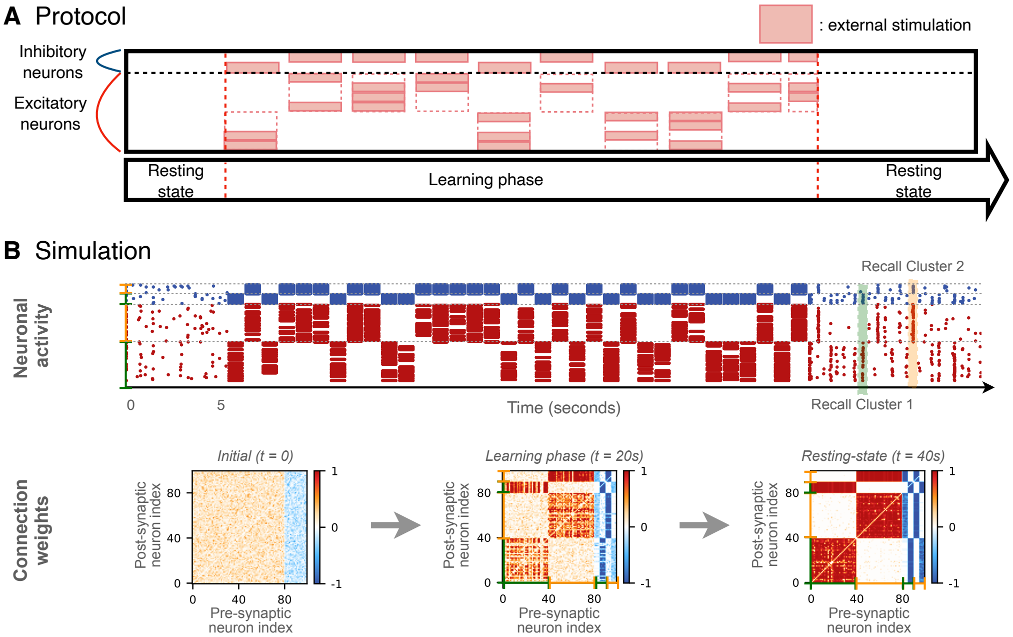

Fig A. Learning of 2 stimuli with randomly stimulated neurons within each distinct population. (A) Stimulation protocol for a network of $N = 100$ neurons entrained with $M = 2$ stimuli, neurons within each population being randomly selected. **(B)** Simulation and learning results. Connectivity matrices show the evolution of the synaptic weights leading to the emergence of two modules. The raster plot shows the simulation for the three stages: initial resting phase, entrainment stage and the post-learning neuronal activity characterized by spontaneous recall events of P_1 neurons (green shadow) and P_2 neurons (orange shadow).
