# Supplementary material for: Emergence and maintenance of modularity in neural networks with Hebbian and anti-Hebbian inhibitory STDP
Source: PLoS Comput Biol. 2025 Apr 22;21(4):e1012973. doi: 10.1371/journal.pcbi.1012973 (PMC12054933; doi:10.1371/journal.pcbi.1012973)
Supplement: S3 Text — (PDF) [file pcbi.1012973.s003.pdf]

S3 Text. Random stimulation values.

This alternative protocol is again analogous to that of experiment of Fig 1D of the main text. The only difference lies in the fact that when a population is stimulated (excitatory and inhibitory neurons), the neurons within it receive inputs of random amplitude (i.e. inducing firing activity between 50 and 100 Hz). The results obtained are described in Fig A. We observe very similar results to those in S2 Text. However in this case, the weight connections seem stronger than in the previous experiment. As a result, the spontaneous recalls appear to be more visible in the dynamics of raster plot. In addition to the conclusions made in the previous study, this experience allows us to conclude that the spatio-temporal correlations of the applied inputs are more impactful on the formation of the modular structures than the intensities of these same inputs. Indeed, the firing frequency induced by the inputs applied to the neurons necessarily has an impact on the encoding of information and the construction of memory as can be seen here. Nevertheless, compared with the previous experiment where the neurons received the same inputs but at times that were not necessarily correlated, the structure is much better learned in the current case.

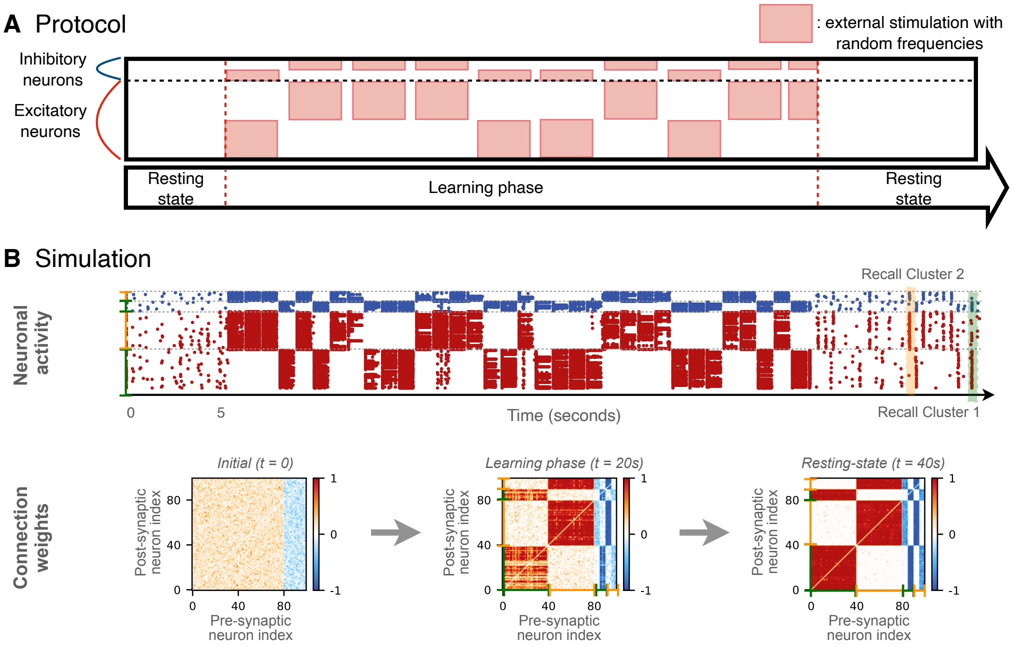

Fig A. Learning of 2 stimuli with random frequencies. (A) Stimulation protocol for a network of $N = 100$ neurons entrained with $M = 2$ stimuli of random amplitude. **(B)** Simulation and learning results. Connectivity matrices show the evolution of the synaptic weights leading to the emergence of two modules. The raster plot shows the simulation for the three stages: initial resting phase, entrainment stage and the post-learning neuronal activity characterized by spontaneous recall events of P_1 neurons (green shadow) and P_2 neurons (orange shadow).
