# Supplementary material for: Emergence and maintenance of modularity in neural networks with Hebbian and anti-Hebbian inhibitory STDP
Source: PLoS Comput Biol. 2025 Apr 22;21(4):e1012973. doi: 10.1371/journal.pcbi.1012973 (PMC12054933; doi:10.1371/journal.pcbi.1012973)
Supplement: S4 Text — (PDF) [file pcbi.1012973.s004.pdf]

S4 Text. Stability of four structural modules.

The reported numerical experiments analyse the limiting cases concerning the stability of four structural modules in absence of any stimulation. In Fig AA, we consider the case where each population contains only one Hebbian and one anti-Hebbian inhibitory neuron (i.e. a total of $N_I = 2 \times 4 = 8$ inhibitory neurons). This arrangement corresponds to the upper limit for the number of inhibitory neurons needed to maintain 4 independent memory items, represented by the red line in Fig 4B of the main text. We observe that these conditions are sufficient for each cluster to present distinct spontaneous recall in neuronal activity.

In Fig AB, we break this limit by allocating only an Hebbian inhibitory neuron to the population P_1. We find that even if other populations are correctly recalled, memory recall of cluster 1 can occur at similar instant to that of the other clusters. Indeed, during recall, population P_1 does not inhibit the activity of the other populations, letting them activate. This has a direct effect on the consolidation process, where simultaneous recall of two memories patterns tends to induce their structural merging.

In Fig AC, we perform an opposite test, assigning only an anti-Hebbian inhibitory neuron to the population P_1. In that case, we observe a very short period of spontaneous activity with recalls from the other population. However, once population P_1 becomes active, it totally dominates the others, inhibiting them. These results are very similar to those observed in Fig 1B. Indeed the absence of feedback inhibition provided by Hebbian inhibitory neuron, prevents P_1 activity to be regulated. Ultimately, this has no direct impact on the long-term maintenance of memory items in the weight matrix since their activity is suppressed. Nevertheless, this causes issues in the processing of stored information, since the network is blocked in an abnormal state.

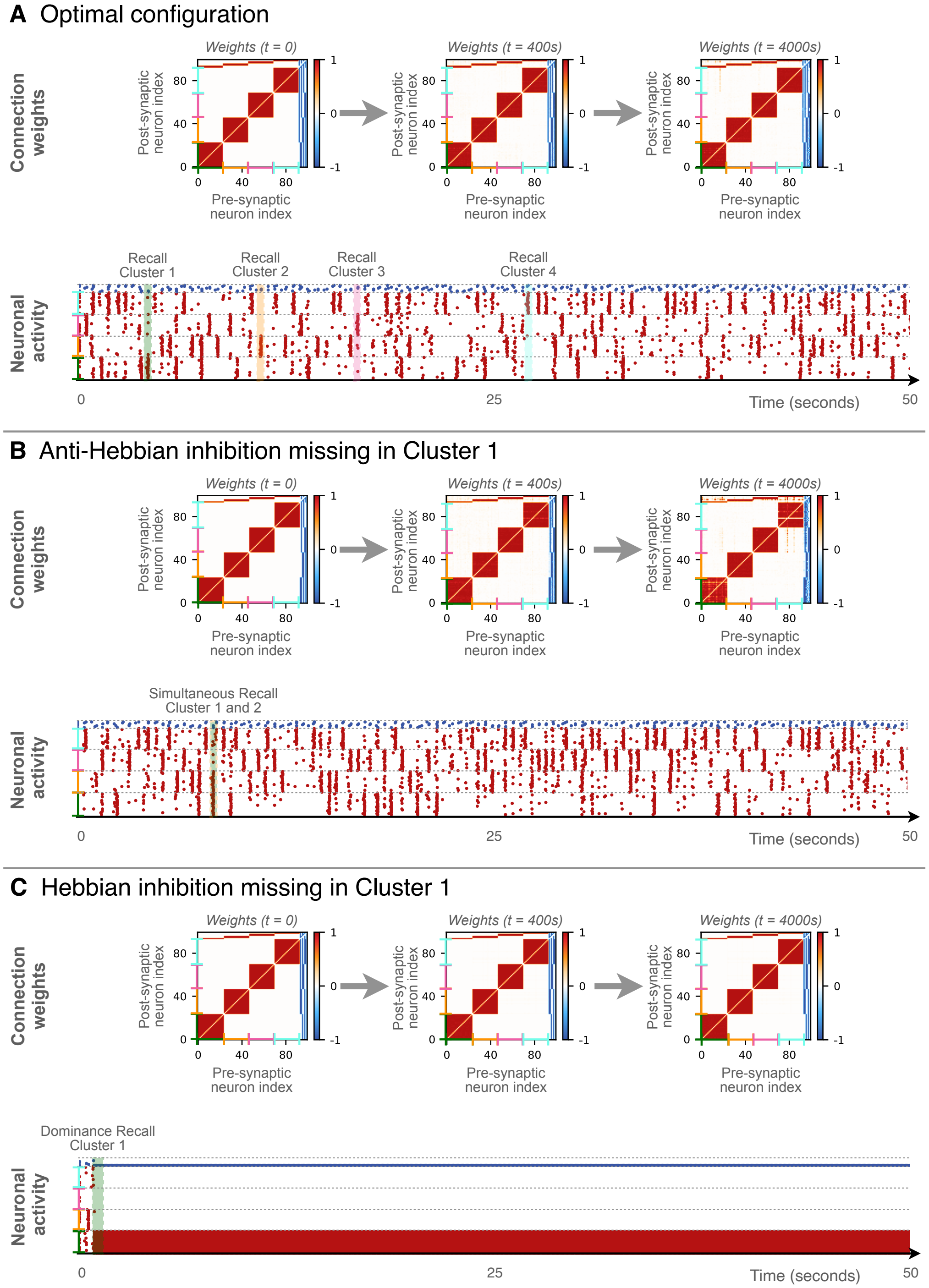

Fig A. Evolution of a network initially made of four structural modules in absence of any stimulation. (A) Network of 92 excitatory neurons and 8 inhibitory neurons (one Hebbian and one anti-Hebbian in each cluster). **(B)** Network of 93 excitatory neurons and 7 inhibitory neurons (only an Hebbian in cluster 1). **(C)** Network of 93 excitatory neurons and 7 inhibitory neurons (only an anti-Hebbian in cluster 1). In each case, we study the stability of the organization from a structural and dynamical point of view. In each panel, the connectivity matrices show the evolution of the synaptic weights and the raster plot shows the neuronal activity. The green, orange, pink and cyan brackets and shadows represent clusters 1, 2, 3 and 4.
