# Supplementary material for: Emergence and maintenance of modularity in neural networks with Hebbian and anti-Hebbian inhibitory STDP
Source: PLoS Comput Biol. 2025 Apr 22;21(4):e1012973. doi: 10.1371/journal.pcbi.1012973 (PMC12054933; doi:10.1371/journal.pcbi.1012973)
Supplement: S5 Text — (PDF) [file pcbi.1012973.s005.pdf]

S5 Text. Four overlapping stimuli.

In this alternative protocol, we reproduce the experiment of Fig 6 of the main text but considering $M = 4$ stimuli which share 8 neurons. The results obtained are described in Fig A. We obtain similar results as in main text with the formation of four modules in the connectivity matrix as expected, together with the formation of hubs which are here connected (incoming and outgoing connections) with the four clusters. Regarding the dynamics in raster plot, the resting-state activity is comparable that observed in Fig 6. We identify different types of spontaneous recalls involving one of the four clusters alone, recalls of one cluster accompanied by the hubs, or recall events of hubs alone. This experiment again highlights the richness of the different dynamics that the network can display and maintain for a certain period of time.

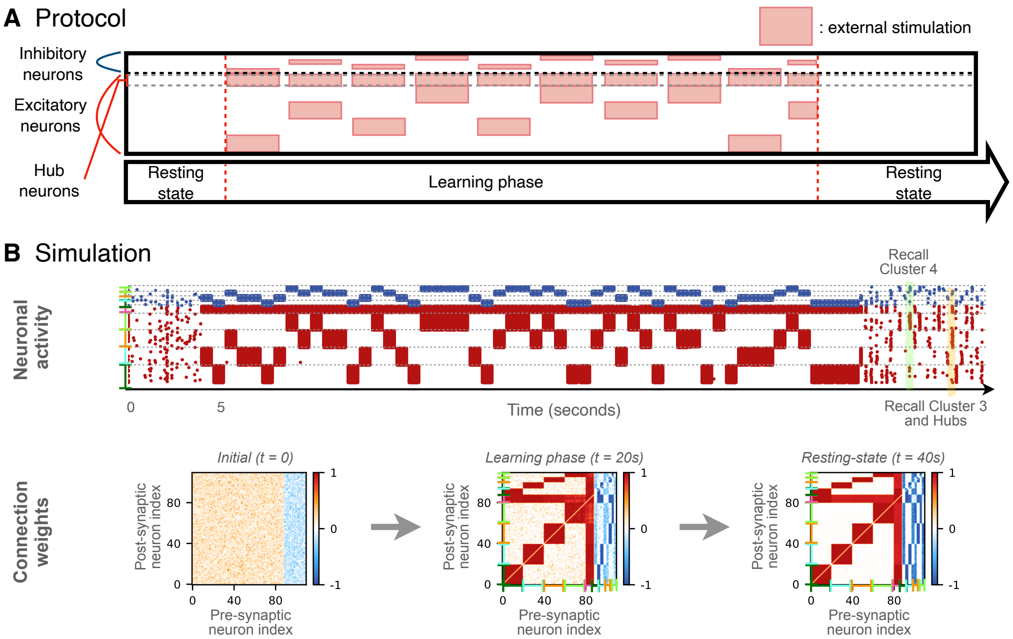

Fig A. Learning of 4 overlapping stimuli. (A) Stimulation protocol for a network of $N = 110$ neurons entrained with $M = 4$ stimuli that share 8 excitatory neurons. **(B)** Simulation and learning results. Connectivity matrices show the evolution of the synaptic weights leading to the emergence of four modules which overlap over 8 hub neurons. The raster plot shows the simulation for the three stages: initial resting phase, entrainment stage and the post-learning neuronal activity characterized by a variety of spontaneous recall events as of P_3 neurons with hubs (orange shadow) or P_4 neurons without hubs (green shadow).
