# Supplementary material for: Emergence and maintenance of modularity in neural networks with Hebbian and anti-Hebbian inhibitory STDP
Source: PLoS Comput Biol. 2025 Apr 22;21(4):e1012973. doi: 10.1371/journal.pcbi.1012973 (PMC12054933; doi:10.1371/journal.pcbi.1012973)
Supplement: S6 Text — (PDF) [file pcbi.1012973.s006.pdf]

S6 Text. Large and sparse networks.

In order to validate the stability of the model for larger network size and sparser connectivity, we apply the protocol reported in Fig 1D of the main text to networks of larger size and with randomly connected neurons, see Fig AA.

Firstly, we consider a network that is initially globally coupled but with random synaptic weights and additive Gaussian noise, of $N = 20000$ neurons, where we still have 80% excitatory and 20% inhibitory neurons. The results of the experiment are reported in Fig AB. Secondly, we consider a random Erdös-Reniy network composed of $N = 1000$ neurons (where we still have a ratio $4 : 1$ for excitatory versus inhibitory neurons), with a probability of 50% of possible directed connections between neurons. In this case, we omit the additive Gaussian noise to check whether the random and sparse connectivity introduces sufficient heterogeneity in the synaptic inputs of the neurons to induce an asynchronous irregular behaviour. The results of this further experiment are reported in Fig AC.

Qualitatively, we obtain the same results as in Fig 1D, with the formation of two modular structures in the weight matrix joined to spontaneous recalls of the two different memories during the post-learning phase. The main differences are indeed observable in this regime. In Fig AB, due to the size of the network it is evident that a large number of neurons fires randomly and independently, this renders more difficult to identify the spontaneous recall events. However, they still take place and the memory should be consolidated on the long term as shown in Fig 5 of the main text.

In the case of the sparse network, the activity during the resting state also presents an irregular activity of the neurons despite the dynamical behaviour is deterministic. However, also in this case, as shown in Fig AC, the modular structures emerge in the weight matrix. One should notice that the orange color in the weight matrix at $t = 50$ seconds is due to the fact that 50% of the neurons are disconnected and do not reflect to the actual value of the weights, that have the same values as in Fig AC.

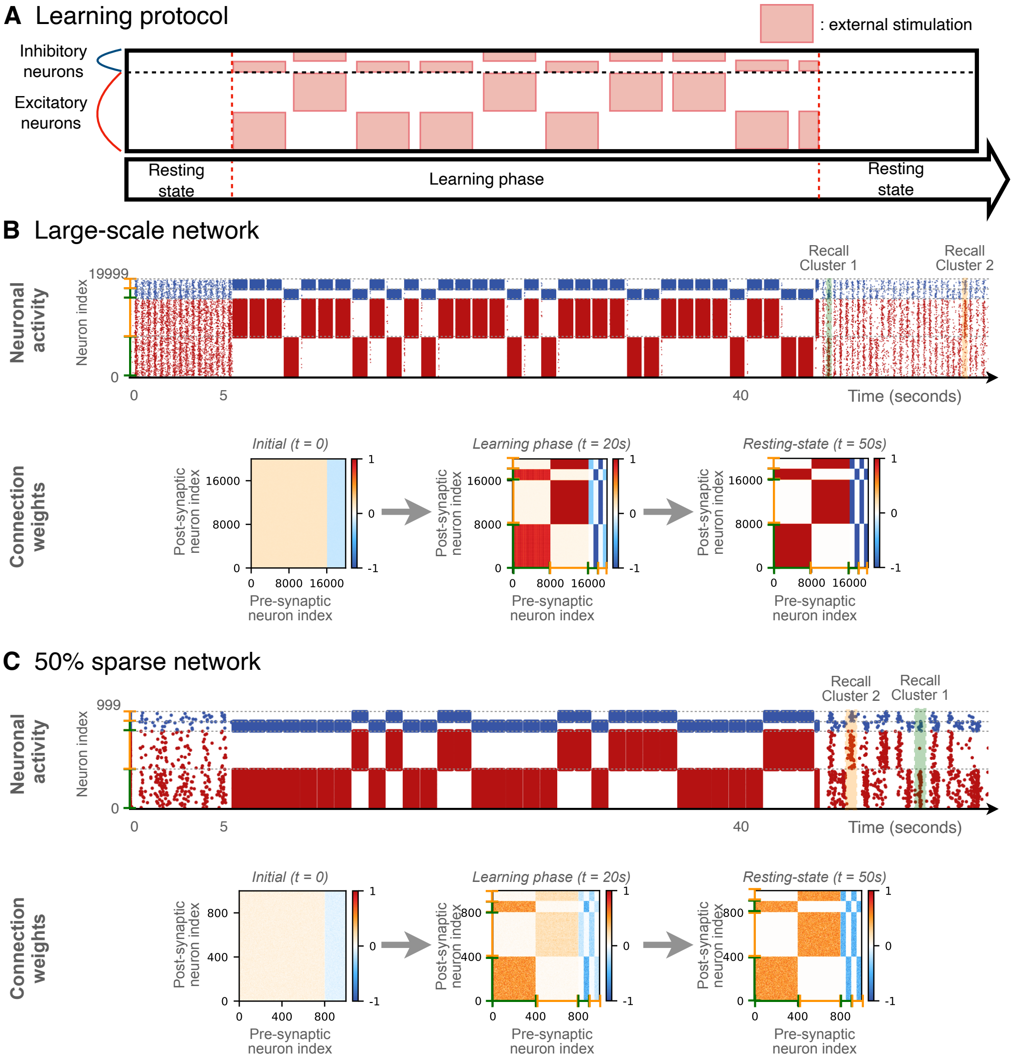

Fig A. Model validation in larger and sparser networks. (A) Stimulation protocol for a network entrained with $M = 2$ stimuli. **(B)** Simulation and learning results for $N = 20000$ neurons. Connectivity matrices show the evolution of the synaptic weights leading to the emergence of two modules. The raster plot shows the simulation for the three stages: initial resting phase, entrainment stage and the post-learning neuronal activity characterized by spontaneous recall events of P_1 neurons (green shadow) and P_2 neurons (orange shadow). **(C)** Simulation and learning results for $N = 1000$ neurons and 50% sparse connection without additive noise terms. Connectivity matrices show the evolution of the synaptic weights leading to the emergence of two modules. The lighter color is due to the sparsity of the connections, but the numerical values are the same as in panel (B). The raster plot shows the simulation for the three stages: initial resting phase, entrainment stage and the post-learning neuronal activity characterized by spontaneous recall events of P_1 neurons (green shadow) and P_2 neurons (orange shadow).
