# Supplementary material for: Emergence and maintenance of modularity in neural networks with Hebbian and anti-Hebbian inhibitory STDP
Source: PLoS Comput Biol. 2025 Apr 22;21(4):e1012973. doi: 10.1371/journal.pcbi.1012973 (PMC12054933; doi:10.1371/journal.pcbi.1012973)
Supplement: S7 Text — (PDF) [file pcbi.1012973.s007.pdf]

S7 Text. Storing the maximum number of items.

Here, we reproduce the experiment of Fig 4A of the main text, but considering $M = 33$ stimuli. This limiting case corresponds to the maximum capacity of items that can be learned and recalled for a network of $N = 100$ neurons (marked by the star symbol in Fig 4B). This implies considering a network composed of $N_I = 66$ inhibitory neurons (with 33 anti-Hebbian and 33 Hebbian). Except these differences in the initial conditions, the network is trained as usual.

This protocol leads to the formation of 33 memories, where each item is composed of an excitatory neuron, a Hebbian and an anti-Hebbian inhibitory neuron, as shown in the connectivity matrix and in the diagram of Fig AB. Consequently, each anti-Hebbian inhibitory neuron projects to all the other clusters of neurons. Concerning the post-learning activity, given the small size of the clusters now, it is difficult to distinguish individual memory recalls with clarity. Nevertheless, the asynchronous dynamics of the network at rest tends to confirm that all excitatory neurons have a decorrelated activity. To visualize this, we estimated the instantaneous Kuramoto order parameter R throughout the simulation. During the post-training resting state the system is essentially desynchronized, since $R \simeq 0.1 \simeq 1/\sqrt{N}$, as expected in an asynchronous system made of $N = 100$ neurons/oscillators due to the central limit theorem. On the contrary, before the training phase the system was partially synchronized, since $R > 0.6$. We have also defined a normalized spike count associated to a certain memory item

$$\rho(t) = \frac{n_m^{sp}(t)}{n^{sp}(t)}$$

where the spike count associated to the neurons related to a given memory item $n_m^{sp}(t)$ is divided by the total number of spikes emitted in the network $n^{sp}(t)$. By computing $\rho(t)$ for the memory highlighted in purple in the figure, we can identify clear recalls when $\rho = 1$, meaning that the spikes only come from the neurons associated to the recalled memory.

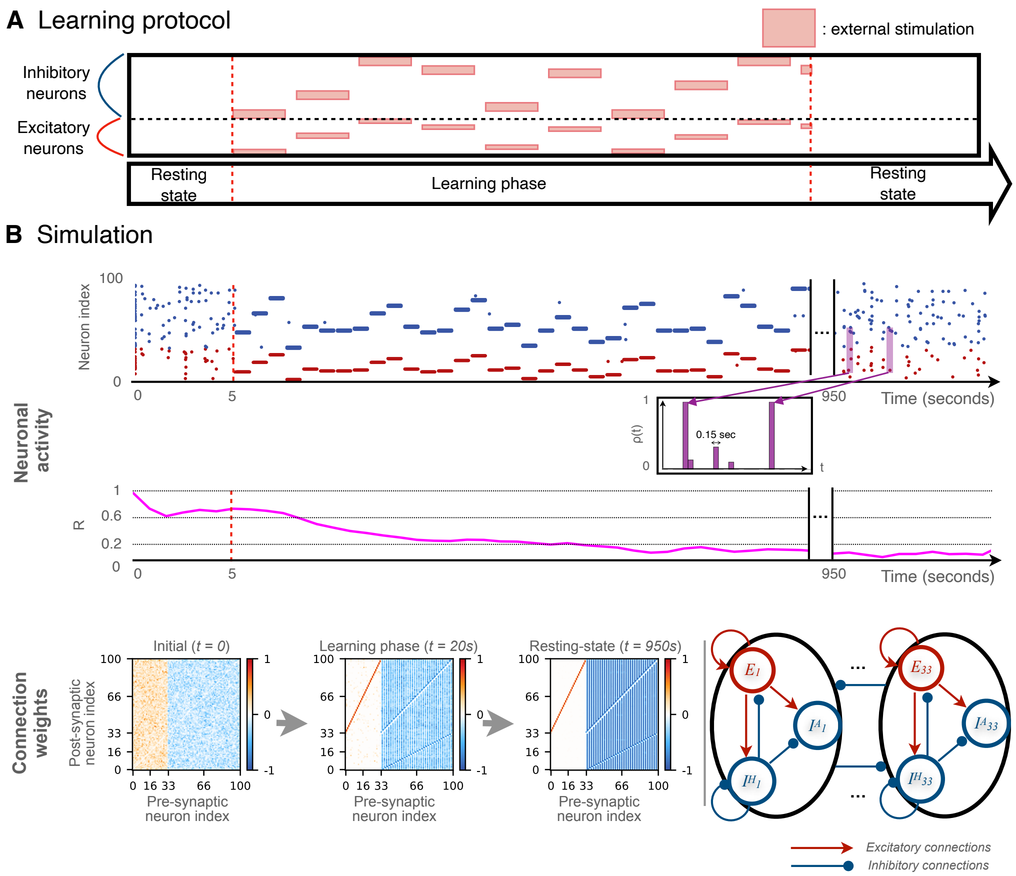

Fig A. Maximum network capacity. (A) Stimulation protocol for a network of $N = 100$ neurons entrained with $M = 33$ stimuli. **(B)** Simulation and learning results. Connectivity matrices show the evolution of the synaptic weights leading to the emergence of 33 modules. The final configuration of the connection weights is shown schematically on the right. The raster plot shows the simulation for the three stages: initial resting phase, entrainment stage and the post-learning neuronal activity characterized by a variety of spontaneous recall events of the memories. The recalls of one memory are highlighted in purple with its normalised spike count $\rho(t)$ for bins of 0.15 sec. The instantaneous Kuramoto order parameters R of the network throughout the simulation time is displayed bellow.
