# Supplementary material for: Emergence and maintenance of modularity in neural networks with Hebbian and anti-Hebbian inhibitory STDP
Source: PLoS Comput Biol. 2025 Apr 22;21(4):e1012973. doi: 10.1371/journal.pcbi.1012973 (PMC12054933; doi:10.1371/journal.pcbi.1012973)
Supplement: S8 Text — (PDF) [file pcbi.1012973.s008.pdf]

S8 Text. Stability of the stored items for increasing network sizes.

By following [1], we have analysed the stability of the intra- and inter-cluster weights for increasing system sizes and numbers of memory items (essentially restricted to $N = 100$ neurons and $M = 2$ memories in the main text). We have here adopted two protocols. In the first, the number of memory items is fixed to $M = 10$. In the other, M grows proportionally with N, in this way the number of neurons associated to each memory clusters remains the same. In both cases we considered three different system sizes, namely, $N = 200$, 500, 1000 and 2000 neurons. The results obtained for $M = 10$ and by varying N are shown in Fig A, while those corresponding to $M = N/100$ are reported in Fig B.

As a general result, we observe that the intra-cluster weights essentially remain constant over time independently of N for both protocols, indicating that the internal stability of the formed clusters is independent of the system size. On the other hand, for the protocol shown in Fig A, we observe a tendency for the inter-clusters weights to grow/decrease over time during the post-learning phase, ultimately leading to a merging of the stored memory items. This can be explained by the fact that reducing network size, reduces the number of inhibitory neurons allocated to each memory, decreasing their stability. Indeed, even if the configurations are sufficient to guarantee coherent reactivations (see S7 Text), less inhibition increases the probability of incoherent spikes due to network variability. However, the rate at which the inter-cluster weights modify strongly decreases with N, indicating that for larger system sizes the memories remains stable for longer times.

Finally, by considering the situation where the ratio M/N is maintained constant as in [1], all intra- and inter-cluster weights remain essentially constant over 4 hours of post-learning evolution, displaying no drift in their values. By employing this second protocol we can extrapolate the memory capacity characterized in the main text for $N = 100$ in Fig 4B to networks of larger size.

A N = 2000 neurons, M = 10 memories

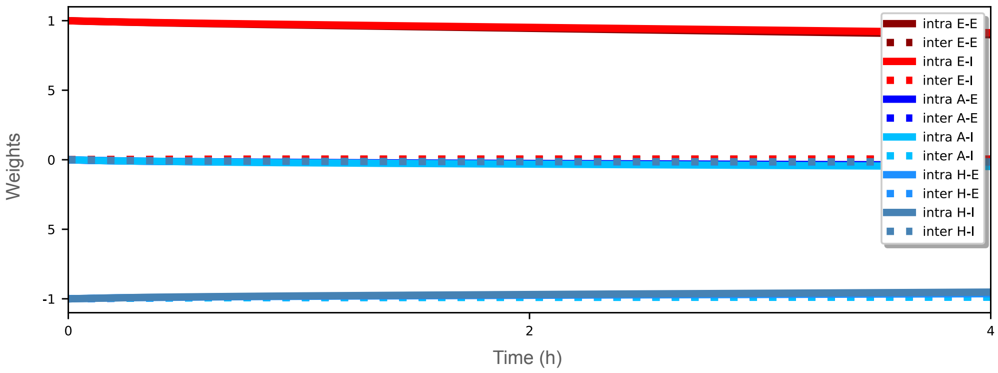

B N = 1000 neurons, M = 10 memories

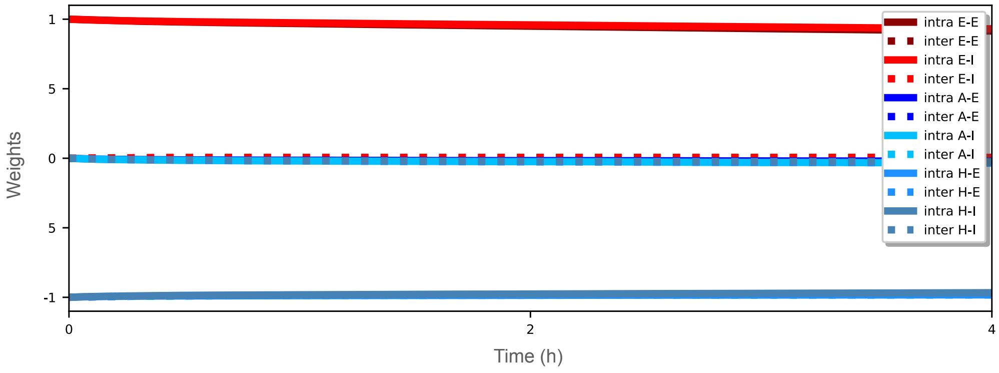

C N = 500 neurons, M = 10 memories

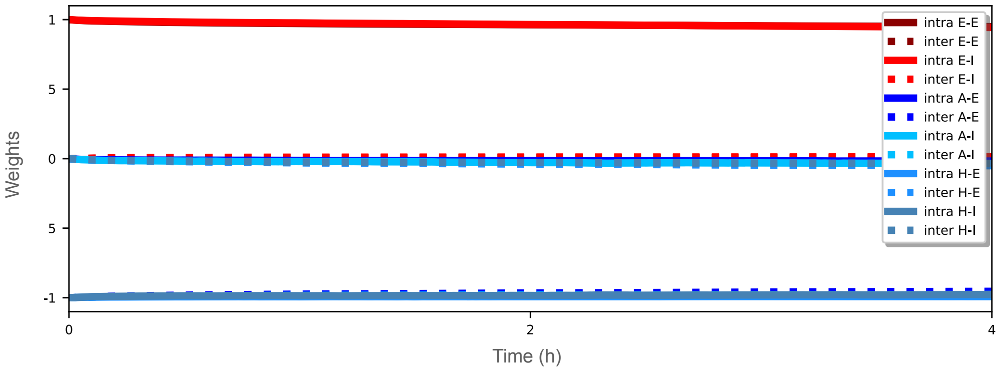

D N = 200 neurons, M = 10 memories

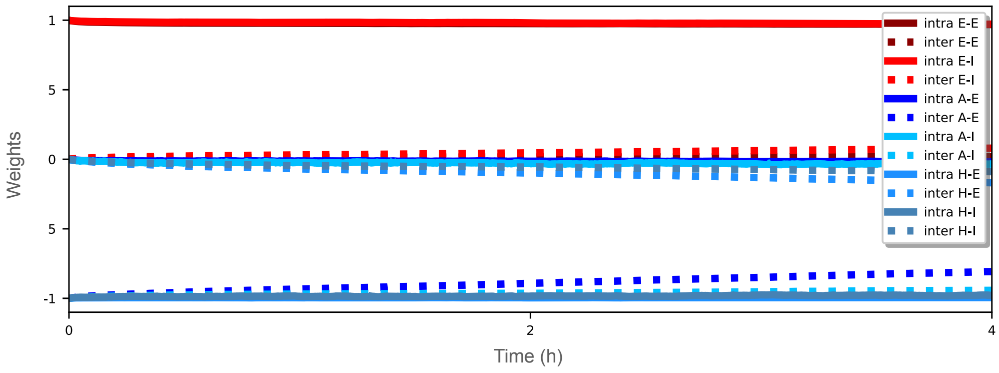

Fig A. Stability of the network connections scaling up the network size. Post-learning evolution of mean intra- (solid lines) and inter- (dashed line) clusters weights for excitatory to excitatory (E-E dark red), excitatory to inhibitory (E-I red), anti-Hebbian inhibitory to excitatory (A-E dark blue), anti-Hebbian inhibitory to inhibitory (A-I cyan), Hebbian inhibitory to excitatory (H-E blue) and Hebbian inhibitory to inhibitory (H-I steel blue) connections; for **(A)** $N = 2000$ neurons, **(B)** $N = 1000$ neurons, **(C)** $N = 500$ neurons, and **(D)** $N = 200$ neurons, all trained with $M = 10$ stimuli.

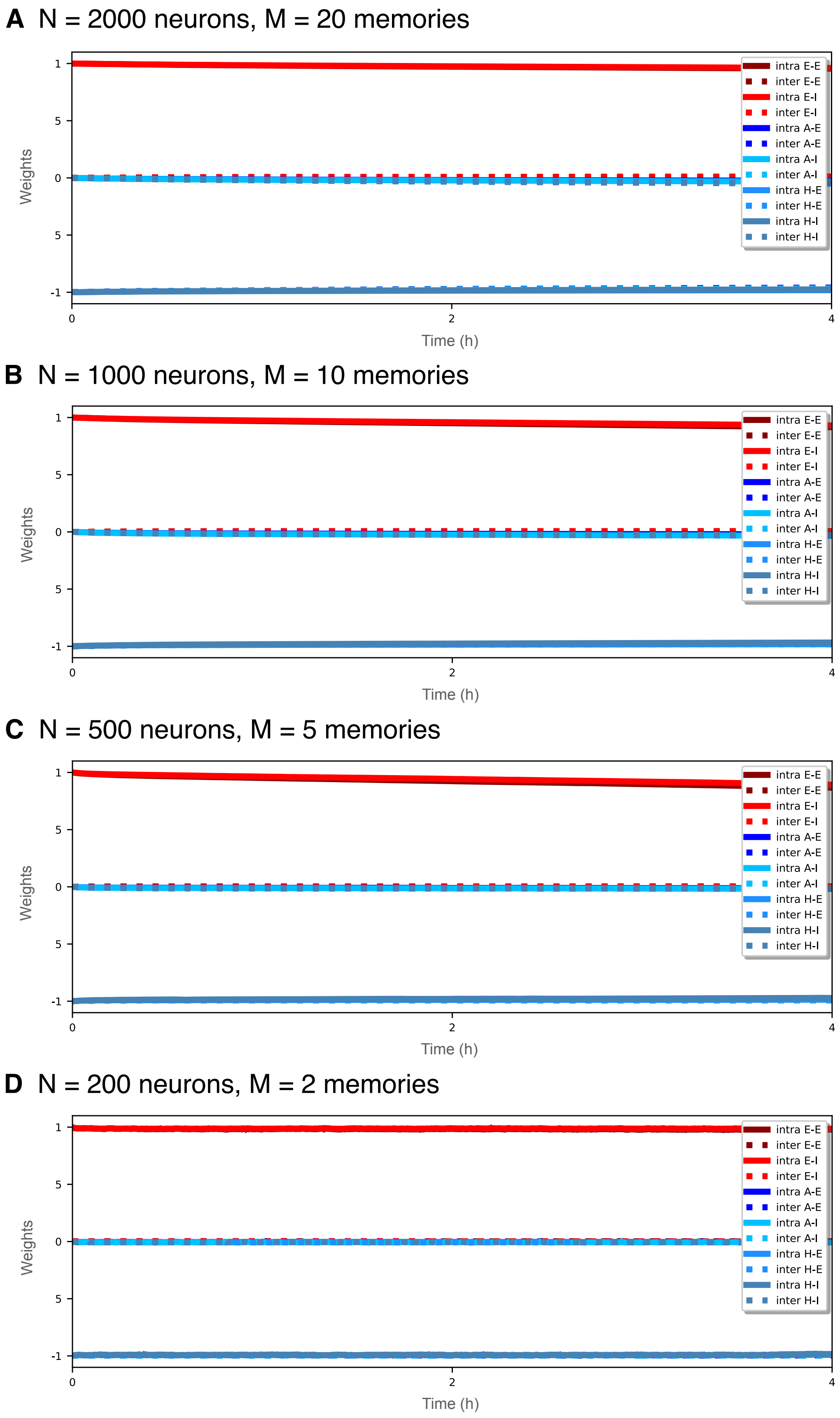
A N = 2000 neurons, M = 20 memories
B N = 1000 neurons, M = 10 memories
C N = 500 neurons, M = 5 memories
D N = 200 neurons, M = 2 memories
Weights
Time (h)
1
5
0
5
-1
0
2
4
intra E-E
inter E-E
intra E-I
inter E-I
intra A-E
inter A-E
intra A-I
inter A-I
intra H-E
inter H-E
intra H-I
inter H-I

Fig B. Network connections stability scaling up M proportionally to N. Post-learning evolution of mean intra- (solid lines) and inter- (dashed line) clusters weights for excitatory to excitatory (E-E dark red), excitatory to inhibitory (E-I red), anti-Hebbian inhibitory to excitatory (A-E dark blue), anti-Hebbian inhibitory to inhibitory (A-I cyan), Hebbian inhibitory to excitatory (H-E blue) and Hebbian inhibitory to inhibitory (H-I steel blue) connections; for **(A)** $N = 2000$ neurons, **(B)** $N = 1000$ neurons, **(C)** $N = 500$ neurons, and **(D)** $N = 200$ neurons, trained respectively with $M = 20$, $M = 10$, $M = 5$ and $M = 2$ stimuli.

References

1. Yang X, La Camera G. Co-existence of synaptic plasticity and metastable dynamics in a spiking model of cortical circuits. PLOS Computational Biology. 2024;20(7):e1012220.
