# Supplementary material for: Emergence and maintenance of modularity in neural networks with Hebbian and anti-Hebbian inhibitory STDP
Source: PLoS Comput Biol. 2025 Apr 22;21(4):e1012973. doi: 10.1371/journal.pcbi.1012973 (PMC12054933; doi:10.1371/journal.pcbi.1012973)
Supplement: S9 Text — (PDF) [file pcbi.1012973.s009.pdf]

S9 Text. Estimation of the time needed to forget a recall event.

The rate of change of the synaptic weights depends on several factors: the current value of the weight $w_{ij}(t)$, the number of neurons spiking at a given time t, and the temporal precision of their spikes. Therefore, it is non-trivial to quantify the precise amplitude of the change of weights at all times. If a synapse is already at its maximum weight capacity, a potentiation (due to a recall) has almost no impact whereas the absence of recalls leads to more impactful depression (see soft bound functions in Methods, Figs 7D and 7E).

We can derive an approximation for the impact of a recall on a synaptic weight—compared to a forgetting epoch—by inspecting closer the plasticity function (see Fig 7A). For uncorrelated spikes during an asynchronous irregular firing epoch (i.e. for $|\Delta t| > 0.5$), the weights depress proportionally to $\Lambda(\Delta t) \approx -0.1$ due to the forgetting term. On the other hand, if the spikes are fully synchronized (i.e. $\Delta t = 0$), their increase is proportional to $\Lambda(\Delta t) = A_+ - A_- - f = 2.347 - f$. Thus, by considering an average weight of $w_{ij} = 0.5$ (where $\tanh(\lambda(1 - w_{ij})) = \tanh(\lambda w_{ij}) \approx 1$) Eq. 10 becomes:

$$\frac{[w_{ij}(t^+) - w_{ij}(t^-)]}{\gamma_l} \simeq \begin{cases} 2.347 - f, & \text{for synchronized spikes during a recall} \;, \\ -f, & \text{for uncorrelated spikes during AI state} \;. \end{cases} \tag{S1}$$

The ratio of these two (absolute) values for potentiation and depression for M stimuli and $f = f_0/M$ is: $\frac{2.347}{f_0}M - 1 = 11.735M - 1$, where $f_0 = 0.2$, and it gives an estimate of the number of uncorrelated spikes that would be required to lose the acquired potentiation. The number of these spikes grows linearly with M. By considering an average firing rate of 2 Hz for the uncorrelated firings, this implies that a period of $\simeq 11$ seconds would be required to forget the contribution of a single recall event for $M = 2$, that will grow to $\simeq 58$ seconds for $M = 10$. The prolongation of this time window proportionally to M will allow to all the stored memory items to be randomly recalled before they are forgotten.
